# Health-Related Quality of Life and Utility Scores in People with Mental Disorders: A Comparison with the Non-Mentally Ill General Population

**DOI:** 10.3390/ijerph110302804

**Published:** 2014-03-07

**Authors:** Amélie Prigent, Ane Auraaen, Blaise Kamendje-Tchokobou, Isabelle Durand-Zaleski, Karine Chevreul

**Affiliations:** 1 AP-HP, URC Eco Ile-de-France, 1, Place du Parvis Notre Dame, Paris F-75004, France; E-Mails: ane.auraaen@urc-eco.fr (A.A.); isabelle.durand-zaleski@sap.aphp.fr (I.D.-Z.); karine.chevreul@urc-eco.fr (K.C.); 2 University Paris Diderot, Sorbonne Paris Cité, ECEVE, UMRS 1123, 10 avenue de Verdun, Paris F-75010, France; 3 Inserm, ECEVE, U1123, 10 avenue de Verdun, Paris F-75010, France; 4 Fondation FondaMental, French National Science Foundation, 40 rue de Mesly, Créteil F-94000, France; 5 University Paris Est, Faculty of Medicine, IFR10, 8, rue du Général Sarrail, Créteil F-94000, France; 6 Centre Hospitalier Départemental Georges Daumézon, 1 route de Chanteau, Fleury-les-Aubrais F-45400, France; E-Mail: akamendje@gmail.com; 7 AP-HP, Henri Mondor University Hospitals, Department of Public Health, 51 avenue du Maréchal de Lattre de Tassigny, Créteil F-94000, France

**Keywords:** mental disorders, health-related quality of life, utility scores, general population

## Abstract

There is a lack of comparable health-related quality of life (HRQoL) and utility data across all mental disorders and all inpatient and outpatient settings. Our objective was to investigate the HRQoL and utility scores of people with mental disorders in France, treated in outpatient and inpatient settings, and to identify the HRQoL and utility score losses attributable to mental disorders compared to the non-mentally ill general population. A cross-sectional survey was conducted to assess HRQoL (SF-12) and utility scores of patients with mental disorders and followed in four psychiatric sectors in France. Scores were described by demographic and clinical characteristics and were then adjusted on age and gender and compared with those of the non-mentally ill general population. Median HRQoL and utility scores were significantly lower in patients with mental disorders than in the non-mentally ill general population; median differences amounted to 5.4 for the HRQoL physical score, to 11.8 for the HRQoL mental score and to 0.125 for the utility score. Our findings underscore the negative impact of mental disorders on HRQoL in France and provide a baseline to assess the global impact of current and future organizational changes in the mental health care system.

## 1. Introduction

Health-related quality of life (HRQoL), which allows quantification of the impact of a disease on an individual’s functioning and well-being, is increasingly used as an important outcome measure in healthcare to support both policy and clinical decision-making [1]. Its measurement, in addition to prevalence and mortality assessments, is essential to inform public health policies by taking into account the relative impact of different conditions [2] as well as to evaluate treatments, interventions or health care organizations [3]. Among a wide range of instruments developed to assess HRQoL, generic measures allow comparisons of distinct conditions as they can be applied across all populations, irrespective of the disease [4]. They assess individuals’ perception of their HRQoL through a set of questions and may be summarized as one or several scores that directly reflect the respondents’ HRQoL. Some measures may also be translated into a single-dimensional utility score that reflects the general population preferences for the health states assessed by the instrument. In these cases, utility scores are obtained through a general population survey in which valuations of health states are elicited from participants according to their preferences using, for instance, the standard gamble or the time trade-off methods [5]. These scores are commonly used in economic studies as a criterion for resource allocation [2,6]. HRQoL scores, directly reflecting individual respondents’ perceptions, and utility scores, which reflect population preferences, may thus be used in a complementary manner to inform policy and clinical decision-making.

Mental disorders often impair functioning in several areas of life resulting in unhappiness and suffering that may affect HRQoL [7]. Given the emphasis on patient-centred, integrated care to address mental health care needs of patients constituting a heterogeneous group and often suffering from multiple mental disorders [8], it is important to globally measure the HRQoL loss due to mental disorders across all disorders and all treatment settings. The resulting data would constitute a decision support to improve mental health care and to determine whether changes to the law or in mental health care organization are having a positive or negative impact on patients’ HRQoL. 

However, there is a lack of comparable HRQoL data across all mental disorders as well as across all treatment settings (inpatient and outpatient). In fact, although surveys in the general population may encompass several mental disorders, hospitalized patients are most often unable to participate [9,10]. By contrast, clinical surveys may include hospitalized patients but often focus on specific mental disorders, mainly mood disorders and schizophrenia, and/or on specific interventions or treatment settings [11,12,13]. Moreover, most of the studies consider raw HRQoL and utility scores that can only be assessed by reference to a theoretical state of perfect health. However, to accurately inform decisions on mental health care organization, assessment should be done by reference to the normative values for the general population without mental disorders, which would allow identification of the share of HRQoL and utility losses due to mental disorders.

Our objective was to investigate the HRQoL and utility scores of individuals with mental disorders in France treated in outpatient and inpatient settings, to describe the demographic and clinical factors associated with differences in HRQoL and utility scores and to identify the HRQoL and utility score losses attributable to mental disorders compared to the non-mentally ill general population.

## 2. Material and Methods

### 2.1. Study Groups

In order to assess HRQoL of patients with mental disorders, we performed a prospective cross-sectional survey in four mental health care catchment areas, or psychiatric sectors, catering to a population of 266,627 adult inhabitants. Psychiatric sectors are the cornerstone of French public mental health care delivery and provide integrated inpatient and outpatient care that is most often coordinated by a public hospital specialized in mental health; in our case, the Daumézon hospital located in the Loiret department in France. 

The survey was conducted on 47 dates between September 2010 and March 2012 in all inpatient care settings, providing full-time or part-time hospitalizations, and in ambulatory care centers, providing outpatient care on a consultation basis. To avoid bias, all patients present the day of the survey in a given care setting and meeting the inclusion criteria were requested to participate. Inclusion criteria were as follows: patients 18 years old and over and suffering from a mental disorder included in the chapter ‘Mental and behavioural disorders’ from the International Classification of diseases, 10th version (ICD-10), except for organic mental disorders (F00–F09), mental retardation (F70–F79) and disorders of psychological development (F80–F89), which were excluded because of the care specificities of these conditions. Face-to-face interviews were conducted with patients to assess their HRQoL after they had been informed and had agreed to participate. Patients’ demographic and clinical characteristics were collected through interviews with nurses and psychiatrists. Demographic characteristics were identified based on medical records, and clinical characteristics were assessed by the psychiatrists at the time of the interview. Finally, 212 patients were included in our study; they were referred as the Patient-MD group. Ethical approvals for this study were obtained from the advisory committee on data processing in the health domain research (comité consultatif sur le traitement de l'information en matière de recherche dans le domaine de la santé (CCTIRS), the national committee of data processing and freedom (commission nationale de l’informatique et des libertés (CNIL)) and the ethical research committee (comité de protection des personnes (CPP)) Ile-de-France IX. 

In order to compare the HRQoL of individuals with mental disorders with the HRQoL of the non-mentally ill general population, we used data from the French population-based survey on health and disabilities (Enquête Handicap-Santé en ménages ordinaires, HSM) [14] conducted by the National Institute for Statistics. This survey was performed in 2008 and consisted of an interview on health and living conditions and a self-report questionnaire including HRQoL. Details about the survey sampling methodology are available elsewhere [15]. Briefly, a representative sample of the general population living in France was obtained in three steps. First, almost 140,000 households were randomly drawn mainly based on the population census in 2007. Each person living in the selected households (262,963 persons) was asked to respond to a short questionnaire to assess his or her level of disability. Second, individuals were included in the final sample using a probability-based method in order to ensure a sufficient representation of people suffering from disabilities, leading to a sample of 29,931 persons. Third, weights were assigned to each respondent to ensure that the sample was representative of the French general population in terms of living area, gender, age and level of disability, for example. For purpose of our study, only individuals of 18 years old and older and who declared that they had not experienced any episode of mental disorders within the past 12 months were included in order to constitute a general population without mental disorders group comparable with the Patient-MD group. Using the weighting methods developed by the National Institute for Statistics, a group representative of the population of adults in France reporting no mental disorders (45,059,223) consisting of 12,558 persons was identified. 

### 2.2. Measurements

Age, gender and HRQoL were collected for both groups. For individuals in the Patient-MD group, the main diagnosis identified from ICD-10 and the disease severity assessed with the Clinical Global Impression—Severity scale (CGI-S) [16] were collected from the psychiatrist in charge of the patient’s treatment and follow-up. 

HRQoL data used to compare the two groups came from the 12-Item Short-Form Health Survey (SF-12). Although the first version of the 36-Item Short-Form Health Survey (SF-36) [17] was administrated to the Patient-MD group, this questionnaire was not fully integrated into the survey of the non-mentally ill general population group, and thus we had to extract only the questions included in the SF-12 for both groups. The SF-12 is a multipurpose short-form generic measure of health status made up of 12 items taken from the SF-36 [18]. Further, the SF-12 covers eight dimensions of health: physical functioning, role limitations due to physical health problems, bodily pain, general health, vitality, social functioning, role limitations due to emotional problems and mental health. The SF-12 allows calculation of two summary HRQoL scores, the Physical Component Summary (PCS-12) and the Mental Component Summary (MCS-12), and may be translated into utility scores [18]. 

Utility scores were derived from the SF-12 using the Medical Outcome Study Short Form 6 Dimensions (SF-6D (SF-12)) and Brazier’s algorithm [19]. The SF-6D (SF-12) uses seven items from the SF-12 and consists of six dimensions: physical functioning, role limitations, social functioning, pain, mental health and vitality, each with between three to five possible levels of answers, level 1 corresponding to a health state without any limitations. To value utility scores associated with each health state, a survey was undertaken in the United Kingdom general population. Utility scores range from 0.35 to 1.00, with 0.35 representing the worst health state and 1.00 the best [19]. 

### 2.3. Statistical Analyses

In order to evaluate the representativeness of the Patient-MD group, we examined administrative data for all patients in inpatient and/or outpatient care in our study area during the survey period who met the inclusion criteria. These populations were compared in terms of age, gender, main diagnosis and occurrence of full-time hospitalization in the past 12 months using one-sample Student t-tests and binomial proportion tests. The Patient-MD and the non-mentally ill general population groups were described in terms of demographic and clinical characteristics. Then, we analyzed unadjusted HRQoL (PCS-12 and MCS-12) and utility scores in the Patient-MD group. Considering the non-normal distributions of the HRQoL and utility scores, we used a non-parametric approach and, thus, presented the median in addition to the mean and standard deviation. Differences with regard to demographic and clinical characteristics were studied using the Kruskal-Wallis test and the Spearman correlation coefficient. Finally, we compared HRQoL and utility scores between the Patient-MD and the non-mentally ill general population groups. To ensure comparability, the Patient-MD group was weighted to reflect the age and gender distribution of the non-mentally ill general population. Given the non-normal distributions of the HRQoL and utility scores, median scores were compared between groups using non-parametric one-sample sign tests and effect sizes were assessed with Cohen’s *g* and classified as small (0.05 ≤ *g* < 0.15), moderate (0.15 ≤ *g* < 0.25) or large (0.25 < *g*) [20]. In addition, both groups were compared regarding the proportion of respondents in each level of each dimension of the SF-6D (SF-12) using binomial proportion tests. A p-value under 0.05 was considered as statistically significant. Analyses were performed using SAS software version 9.3 for Windows (SAS Institute Inc., Cary, NC, USA).

## 3. Results

### 3.1. Characteristics of the Studied Populations

Of the 212 patients included in the Patient-MD group, 203 had complete data on HRQoL. To assess the representativeness of our sample, we compared the Patient-MD group to the overall population followed in the psychiatric sectors in our study area based on administrative data. The Patient-MD group did not differ significantly in terms of gender and full-time hospitalization within the past 12 months but was older on average (51.1 *vs*. 46.7; *p* < 0.001) and was comprised of a higher proportion of people suffering from schizophrenia, schizotypal and delusional disorders (ICD-10 codes in F2) (8.5% more in the Patient-MD group than in the whole population, *p* = 0.022). The diagnoses with the highest prevalence in the Patient-MD group were mood disorders, followed by schizophrenia, schizotypal and delusional disorders and anxiety disorders. More than half of the patients were considered as severely ill and 41% were hospitalized within the previous 12 months (Table 1). 

Compared to the non-mentally ill general population, patients in the Patient-MD group were older (51.1 years old in average *vs*. 47.5; *p* < 0.001); the age group 18–29 was under-represented in the Patient-MD group whereas the age group 40–69 was over represented. No significant difference was identified with regard to gender (Table 1). 

**Table 1:** Demographic and clinical characteristics of the studied populations.

Demographic and Clinical Characteristics	Patient-MD Group (*N* = 203)		Non-Mentally Ill General Population
	%	*N*	%
Gender, Female	54.2	110	51.8
Ten-year age groups			
18–29	5.9	12	19.8
30–39	13.3	27	18.3
40–49	26.6	54	18.1
50–59	27.1	55	17.4
60–69	16.8	34	11.8
>70	10.3	21	14.7
Main diagnoses			NA
Mental and behavioural disorders due to psychoactive substance use	8.4	17	
Schizophrenia, schizotypal and delusional disorders	32.5	66	
Mood disorders	34.5	70	
Anxiety disorders	13.3	27	
Other mental and behavioural disorders	5.4	11	
Missing	5.9	12	
Disease severity			NA
Mild and moderate (CGI-S scores 2–4)	35.0	71	
Severe (CGI-S scores 5–7)	52.2	106	
Missing	12.8	26	
Full-time hospitalization within the previous 12 months	41.4	84	NA

NA: not applicable.

### 3.2. Unadjusted HRQoL and Utility Scores in the Patient-MD Group

The Kolmogorov-Smirnov test for normality led to rejection of the normality assumption of the distributions for PCS-12 (*p* < 0.010), MCS-12 (*p* = 0.022) and the utility score (*p* < 0.010).

In the Patient-MD group, the median PCS-12 was 46.8 (mean, 45.2; SD, 9.2), the median MCS-12 was 37.7 (mean, 39.1; SD, 11.8) and the median utility score was 0.657 (mean, 0.676; SD, 0.120).

HRQoL and utility scores were systematically higher for male patients than for female patients (Table 2). A negative non-linear relationship was found between age and PCS-12 (*rho* = −0.32, *p* < 0.001) whereas no statistically significant relationships were identified with age for MCS-12 and the utility score (*rho* = 0.14, *p* = 0.054; *rho* = −0.01, *p* = 0.899). Regarding main diagnosis and disease severity, statistically significant differences were only identified for the utility score. The median utility score varied by mental disorder group from 0.626 for people with mood disorders to 0.693 for people with schizophrenia, schizotypal and delusional disorders and was lower for severely ill patients (Table 3). The HRQoL and utility scores significantly differed between patients who had been hospitalized within the 12 past months and those who were not. While the PCS-12 score was lower for non-hospitalized patients, the MCS-12 and utility scores were higher (Table 3). 

**Table 2:** HRQoL and utility scores in the Patient-MD group by gender (*N* = 203).

Gender	PCS-12			MCS-12			Utility Score		
	Mean (SD)	Median	*p*	Mean (SD)	Median	*p*	Mean (SD)	Median	*p*
Male	47.3 (8.9)	49.5	0.002	41.3 (11.0)	42.4	0.011	0.704 (0.114)	0.691	0.001
Female	43.5 (9.2)	43.9		37.2 (12.3)	34.9		0.652 (0.120)	0.640	

**Table 3:** HRQoL and utility scores in the Patient-MD group by clinical characteristics.

Clinical Characteristics	PCS-12				MCS-12				Utility Score		
	Mean (SD)	Median	*p*		Mean (SD)	Median	*p*		Mean (SD)	Median	*p*
Main diagnoses (*N* = 192 *)											
Mental and behavioural disorders due to psychoactive substance use	47.2 (10.7)	49.5	0.293		36.7 (11.2)	31.3	0.072		0.673 (0.098)	0.657	0.037
Schizophrenia, schizotypal and delusional disorders	46.4 (9.4)	49.4			42.4 (10.9)	42.7			0.708 (0.121)	0.693	
Mood disorders	44.0 (8.9)	43.9			36.8 (12.2)	35.4			0.645 (0.122)	0.626	
Anxiety disorders	44.8 (9.6)	45.7			37.8 (13.3)	34.1			0.663 (0.120)	0.629	
Other mental and behavioural disorders	44.8 (9.4)	41.4			38.4 (11.3)	36.6			0.697 (0.104)	0.663	
Disease severity (*N* = 177 *)											
Mild and moderate (CGI-S scores 2–4)	46.7 (8.3)	48.7	0.256		41.2 (11.8)	41.6	0.120		0.706 (0.128)	0.695	0.044
Severe (CGI-S scores 5–7)	44.8 (9.7)	46.1			38.3 (11.9)	37.0			0.663 (0.112)	0.655	
Full-time hospitalization within the previous 12 months (*N* = 203)											
Yes	46.8 (9.2)	48.3	0.045		34.7 (10.8)	33.8	<0.001		0.641 (0.097)	0.626	<0.001
No	44.2 (9.2)	45.8			42.1 (11.6)	42.5			0.701 (0.128)	0.683	

* Twelve patients were excluded from this analysis because of missing values on main diagnosis and 26 because of missing values on disease severity score.

### 3.3. Comparisons with the Non-Mentally Ill General Population

#### 3.3.1. HRQoL and Utility Scores

After adjusting the Patient-MD group on age and gender, the normality assumption was again rejected based on the Kolmogorov-Smirnov test for normality (PCS-12, *p* < 0.010; MCS-12, *p* < 0.010; utility score, *p* < 0.010). We found that HRQoL and utility scores were significantly lower for people suffering from mental disorders than for the non-mentally ill general population (*p* < 0.001). Median differences (mean differences) were 5.4 (4.2) for PCS-12, 11.8 (9.1) for MCS-12 and 0.125 (0.083) for the utility score (Table 4). Effect sizes were large for the three scores (Cohen’s *g* > 0.25). 

**Table 4:** HRQoL and utility scores in the Patient-MD group (adjusted) and in the non-mentally ill general population.

HRQoL and Utility Scores	Patient-MD Group (*N* = 203)		Non-Mentally Ill General Population		Sign Test, *p*	Cohen’s *g*
	Mean (SD)	Median	Mean (SD)	Median		
PCS-12	46.2 (9.1)	48.1	50.4 (9.5)	53.5	<0.0001	0.29
MCS-12	38.8 (12.0)	38.1	47.9 (9.9)	49.9	<0.0001	0.26
Utility score	0.683 (0.121)	0.657	0.766 (0.137)	0.782	<0.0001	0.29

#### 3.3.2. Dimensions of HRQoL Affected

**Figure 1:**
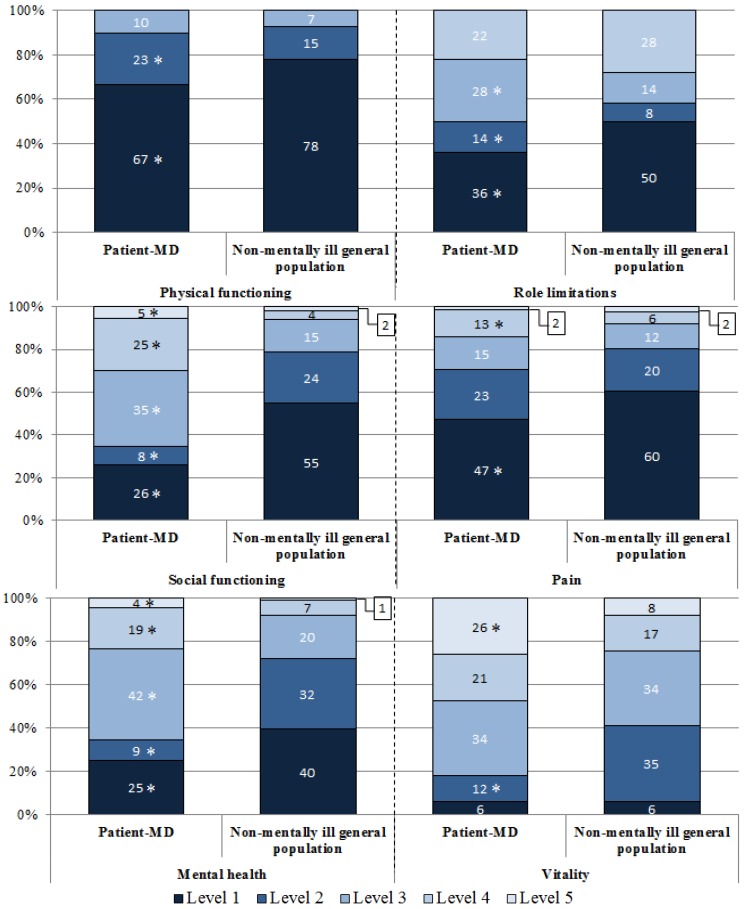
Differences in the distribution for the SF-6D (SF-12) dimensions between the Patient-MD group (*N* = 203) and the non-mentally ill general population.

For the SF-6D (SF-12) dimensions, the most important differences were identified for the dimensions social functioning, mental health and vitality (Figure 1). In fact, in the social functioning dimension, only one-third of the Patient-MD group reported that their health limits their social activities none of the time (level 1) or a little of the time (level 2) compared to four-fifths of the non-mentally ill general population (*p* < 0.001). Only one-third of the Patient-MD group was classified in level 1 or 2 in the mental health dimension (feel downhearted and low none of the time or a little of the time) compared to almost three-quarters in the non-mentally ill general population (*p* < 0.001). For the vitality dimension, differences were found in levels 2 (having a lot of energy most of the time) and 5 (having a lot of energy none of the time), a less important part of people in the patient-MD group reported being in level 2 (one-tenth *vs*. one-third, *p* < 0.001) whereas further people reported being in level 5 (one-quarter *vs*. one-tenth, *p* < 0.001). 

## 4. Discussion and Conclusions

The findings of our study show that, after adjusting for age and gender, HRQoL and utility scores were significantly lower in people being treated for mental disorders in inpatient and/or outpatient care treatment in the psychiatric sectors compared with the non-mentally ill general population. As expected, the gap between groups was greater for the HRQoL mental score than for the physical score. Difference in mean utility scores was significant and amounted to twice the minimally important difference for SF-6D utility scores, estimated at 0.041 [21].

* Statistically significant difference with the non-mentally ill general population at the 5% level population.

Our results highlighted the negative impact that mental disorders have on HRQoL, which is consistent with the findings of other studies focused on specific disorders or treatment settings [9,22]. As utility scores vary depending on the specific instrument used [23], our findings on utility scores are only directly comparable to studies using the SF-6D (SF-12) utility measure. We identified only one study that analyzed median SF-6D (SF-12) utility scores in patients with mental disorders in primary care [24]. The results were consistent with our findings showing that mood disorders had the strongest impact on utility scores as well as with findings of a study that used a different utility measurement [2]. To our knowledge, no study has focused broadly on all mental disorders in both inpatient and outpatient facilities, and thus our overall results are not directly comparable with previous studies. 

Our study has a few limitations. Only patients who were able and willing to participate were included in the Patient-MD group and thus, patients in an acute stage of illness were not represented, which may have led to an overestimation of HRQoL and utility scores. Concerning the general population sample, individuals free from mental disorders were selected on a self-reported basis. Participants in the survey were asked to list all diseases they had suffered from in the previous 12 months. As mental disorders may be under-reported by those suffering from them [25], we may have included individuals with mental disorders in the non-mentally ill general population group and thus probably underestimated the HRQoL and utility score differences between the two groups.

Our survey focused on the public mental health sector but did not take into account HRQoL of patients followed in the private sector (general practitioners, self-employed psychiatrists or private psychiatric hospitals). Additional studies would be necessary to determine the direction of the associated bias. General practitioners or self-employed psychiatrists are more likely to follow people with mood and anxiety disorders [26], which are associated with lower utility scores, but also potentially with being less severely ill, which is associated with higher utility scores. 

We identified the HRQoL and utility score losses due to mental disorders by comparison with a demographically representative population-based sample. However, our results were not adjusted for potential comorbid physical illnesses. This choice is explained by the fact that physical disorders were assessed on a declaratory basis in the general population survey and are not systematically reported and diagnosed in the mental health care facilities in which the Patient-MD group was recruited. Moreover, it has been demonstrated that physical disorders are more prevalent and responsible for 60% of the excess mortality in people suffering from severe mental disorders [27,28]. Individual lifestyle choices contribute to this burden, but psychotropic medications together with disparities in access to health care also explain the higher prevalence of physical comorbidities in people with severe mental disorders [28]. The fact that mental disorders impact physical health, particularly in terms of reduced access to and lower quality of health care, led us to account for these negative impacts in the individual burden attributable to mental disorders. By the same logic, while low socioeconomic status may partly be explained by mental disorders, we did not adjust for socioeconomic status given the variability of this effect among distinct disease categories [29]. 

Even though we used the SF-36 in the Patient-MD group, we had to limit our analysis to the SF-12, and thus to the SF-6D (SF-12), to ensure data comparability with the non-mentally ill general population despite the fact that it defines fewer distinct health states and provides less reliable estimates of the individual level of health than the SF-36 [30]. However, the PCS-12 and MCS-12 were found to lead to the same statistical conclusions as the PCS-36 and MCS-36 respectively [30]. Moreover, use of utility scores derived from SF-6D (SF-12) has been demonstrated to result in little loss of information compared to those derived from the SF-36 [19]. Based on data of the Patient-MD group, utility scores derived from SF-12 and SF-36 respectively were found to be highly correlated (Spearman coefficient correlation, 0.920; *p* < 0.001). Furthermore, in the absence of French utility values associated with SF-6D (SF-12), we used United Kingdom tariffs, which possibly introduced a cultural bias. 

We only provided a short bivariate analysis of patients’ characteristics associated with HRQoL and utility scores in order to better describe our sample. However, complementary multivariate analyses based on appropriate non-parametric approaches and including a complete set of demographic and clinical characteristics, as well as information on types of mental health care received, is necessary in order to fully identify factors associated with HRQoL and utility scores [31]. 

Finally, we only calculated the average burden of mental disorders at an individual level, whereas its calculation at the population level by considering the prevalence of the diseases constitutes part of the relevant information to support policy decision-making [9]. However, we were unable to estimate the burden at population level for France as prevalence data on people suffering from mental disorders and followed by public mental health care settings are unavailable. Given that the average individual burden attributable to mental disorders was 0.083 within our study area and that 2,266 patients had been in treatment in the included settings, the total burden per 100,000 inhabitants was estimated at 70.53 QALYs lost per year. These results are consistent with Saarni *et al.*, 2007, who used the EQ-5D instrument to estimate the annual number of QALYs lost per 100,000 inhabitants for different mental illnesses and found the burden ranged from 298 for dysthymia to 48 for panic disorders. 

Our study has provided data on HRQoL and utility scores for patients suffering from the broad spectrum of mental disorders and receiving treatment in inpatient and/or outpatient settings. HRQoL has been recognized as an important outcome in health care that allows taking into account negative impacts of diseases on functioning and well-being and thus should be considered in the assessment of modifications in the organization of health care services. This is essential in the field of mental health in which numerous challenges remain to better address needs of people suffering from mental disorders [32,33]. Fundamental changes are needed in terms of resource allocation, access to primary care, development of community-based services and legislation to protect human rights [32,33]. Implementations of current and future policies will affect mental health care organization in both inpatient and outpatient care settings involved in the treatment of the full range of mental disorders. If the impact of these evolutions on health care costs and care consumption may be assessed a posteriori using administrative databases, the positive or negative impact on HRQoL, directly affecting patients, cannot be evaluated as HRQoL is not measured in routine clinical practice. Our study, in addition to underscoring the negative impact of mental disorders on HRQoL in France, has produced baseline data that are essential in order to assess the global impact of organizational changes in the mental health care system. 
